# Machine learning for accurate estimation of fetal gestational age based on ultrasound images

**DOI:** 10.1038/s41746-023-00774-2

**Published:** 2023-03-09

**Authors:** Lok Hin Lee, Elizabeth Bradburn, Rachel Craik, Mohammad Yaqub, Shane A. Norris, Leila Cheikh Ismail, Eric O. Ohuma, Fernando C. Barros, Ann Lambert, Maria Carvalho, Yasmin A. Jaffer, Michael Gravett, Manorama Purwar, Qingqing Wu, Enrico Bertino, Shama Munim, Aung Myat Min, Zulfiqar Bhutta, Jose Villar, Stephen H. Kennedy, J. Alison Noble, Aris T. Papageorghiou

**Affiliations:** 1 Institute of Biomedical Engineering, Department of Engineering Science, University of Oxford, Oxford, UK; 2 Nuffield Department of Women’s & Reproductive Health, University of Oxford, Oxford, UK; 3 Intelligent Ultrasound Ltd, Hodge House, Cardiff, CF10 1DY UK; 4 South African Medical Research Council Developmental Pathways for Health Research Unit, Department of Paediatrics & Child Health, University of the Witwatersrand, Johannesburg, South Africa; 5 College of Health Sciences, University of Sharjah, University City, United Arab Emirates; 6 Maternal, Adolescent, Reproductive & Child Health (MARCH) Centre, London School of Hygiene & Tropical Medicine, London, UK; 7 Programa de Pós-Graduação em Epidemiologia, Universidade Federal de Pelotas, Pelotas, Brazil; 8 Programa de Pós-Graduação em Saúde e Comportamento, Universidade Católica de Pelotas, Pelotas, Brazil; 9 Faculty of Health Sciences, Aga Khan University, Nairobi, Kenya; 10 Department of Family & Community Health, Ministry of Health, Muscat, Oman; 11 Departments of Obstetrics and Gynecology and of Global Health, University of Washington, Seattle, WA USA; 12 Nagpur INTERGROWTH-21st Research Centre, Ketkar Hospital, Nagpur, India; 13 School of Public Health, Peking University, Beijing, China; 14 Dipartimento di Scienze Pediatriche e dell’ Adolescenza, Struttura Complessa Direzione Universitaria Neonatologia, Università di Torino, Torino, Italy; 15 Department of Obstetrics & Gynaecology, Division of Women & Child Health, Aga Khan University, Karachi, Pakistan; 16 Shoklo Malaria Research Unit, Mahidol-Oxford Tropical Medicine Research Unit, Faculty of Tropical Medicine, Mahidol University, Mae Sot, Tak Thailand; 17 Center for Global Child Health, Hospital for Sick Children, Toronto, Canada; 18 Oxford Maternal & Perinatal Health Institute, Green Templeton College, University of Oxford, Oxford, UK

**Keywords:** Ultrasonography, Intrauterine growth, Neonatology

## Abstract

Accurate estimation of gestational age is an essential component of good obstetric care and informs clinical decision-making throughout pregnancy. As the date of the last menstrual period is often unknown or uncertain, ultrasound measurement of fetal size is currently the best method for estimating gestational age. The calculation assumes an average fetal size at each gestational age. The method is accurate in the first trimester, but less so in the second and third trimesters as growth deviates from the average and variation in fetal size increases. Consequently, fetal ultrasound late in pregnancy has a wide margin of error of at least ±2 weeks’ gestation. Here, we utilise state-of-the-art machine learning methods to estimate gestational age using only image analysis of standard ultrasound planes, without any measurement information. The machine learning model is based on ultrasound images from two independent datasets: one for training and internal validation, and another for external validation. During validation, the model was blinded to the ground truth of gestational age (based on a reliable last menstrual period date and confirmatory first-trimester fetal crown rump length). We show that this approach compensates for increases in size variation and is even accurate in cases of intrauterine growth restriction. Our best machine-learning based model estimates gestational age with a mean absolute error of 3.0 (95% CI, 2.9–3.2) and 4.3 (95% CI, 4.1–4.5) days in the second and third trimesters, respectively, which outperforms current ultrasound-based clinical biometry at these gestational ages. Our method for dating the pregnancy in the second and third trimesters is, therefore, more accurate than published methods.

## Introduction

Failure to estimate gestational age (GA) accurately remains an important barrier to the provision of evidence-based pregnancy care in many low- and middle-income countries (LMICs)^1^. An accurate estimate of GA is crucial to inform decision-making at the individual level. It is also essential at population level to measure causes of infant morbidity and mortality, such as preterm birth and small for GA (SGA)^2^ - information that is needed for public health strategies to improve health outcomes.

Currently, ultrasound measurement of the fetal crown rump length (CRL) between 11 and 14 weeks’ gestation is the most accurate method to establish GA, i.e., the gold standard. However, many women, especially in LMICs, first seek antenatal care much later in pregnancy because of lack of resources and/or socio-cultural issues^3,4^. Relying on the reported last menstrual period (LMP) to estimate GA is invariably unhelpful due to inaccurate recall of dates, or irregular menstrual cycles, often exacerbated by malnutrition^5^.

Consequently, across the world, GA is mostly determined in the second and third trimesters by measurement of symphysis-fundal height (SFH) or fetal size using ultrasound. Even though ultrasound is more accurate than SFH measurement^6^, biometry-based GA assessment late in pregnancy is fundamentally flawed because it assumes the fetus to have a mean size. By equating fetal size with GA this practice neglects biological variation with two main clinical effects: firstly, increased variation in ‘normal’ fetal size^7^ means that accuracy of GA estimation becomes less reliable as pregnancy advances, so that after 32 weeks’ gestation, dating based on biometry has a prediction interval in excess of ± 2 weeks^7^; and secondly, pathological aberrations of growth become more common as pregnancy advances, and the assumption of average fetal size means biometry-based GA estimation underestimates GA in SGA fetuses and overestimates it in large for GA (LGA) fetuses^8,9^.

Here we propose machine learning as an alternative approach. This is effective for multiple ultrasound image analysis tasks, including image registration^10^, classification^11^ and regression^12,13^. Nevertheless, existing methods that automatically derive biometric measures from standard ultrasound planes result in the same uncertainties as clinical measurement^12^, or limited to GA estimation using a single fetal standard plane^14^ or video^15^.

As the appearance of ultrasound images varies according to GA due to, for example, increased fetal brain gyration, enhanced liver echogenicity, or skeletal ossification as pregnancy advances, we aimed to examine whether machine learning of fetal ultrasound images acquired in the second and third trimesters can generate accurate GA estimates solely using image characteristics, without resorting to any size-based information. This would provide an alternative method for GA estimation for the large number of women globally who first attend an antenatal clinic after 14 weeks’ gestation. Using ultrasound images from two large global ultrasound studies we train and externally validate machine learning models for GA assessment, blinded to the ground truth of GA (based on assessment in the first trimester). All measurements and scale information were removed from ultrasound images and a CNN architecture consisting of modularised convolutional layers characterised with skip connections between modules utlilsed. Thus, fetal ultrasound image appearance is used to estimate GA. Estimation using three standard planes of head circumference (HC), abdominal circumference (AC) and femur length (FL) show a mean absolute error of ±4 days; estimation is within ±7 days of the gold standard in 85% of images in external validation throughout second and third trimesters (13^+0^ to 42^+0^ weeks). Moreover, this accuracy is maintained in the presence of SGA and LGA. The method overcomes the longstanding problem of biometry-based GA estimation in late pregnancy which results in much larger errors due to increasing variability in fetal size, greater absolute scanning error, and a higher incidence of SGA and LGA.

## Results

### Demographics

The average age of women was 27.8, 27.9, and 30.2 years in the training and internal validation set (INTERGROWTH-21st), and external validation set (INTERBIO-21st), respectively. Women enroled in INTERBIO-21st had a higher mean weight and body mass index than those participating in INTERGROWTH-21st and also a higher rate of preterm birth (11.8% versus 4.7 and 2.8% in the training and internal validation datasets from INTERGROWTH-21st), which is expected given the higher risk status. Further demographic information can be found in Table 1.

**Table 1:** Demographics of women included in this study.

	INTERGROWTH-21st (*n* = 4233)		INTERBIO-21st
Characteristic	Training set (*n* = 3809)	Internal validation (*n* = 424)	External validation (*n* = 2433)
Maternal age (years)	27.8 ± 3.8	27.9 ± 3.9	30.2 ± 5.1
Maternal height (cm)	162.2 ± 5.8	162.4 ± 6.0	160.9 ± 7.1
Maternal weight (kg)	61.6 ± 9.2	61.2 ± 9.2	65.2 ± 12.3
Maternal BMI (kg/m^2^)	23.3 ± 3.0	23.0 ± 2.8	25.1 ± 4.2
GA at first visit (weeks)	11.8 ± 1.4	11.8 ± 1.4	11.9 ± 1.3
Nulliparous	2612 (68.6)	292 (69.3)	976 (40.1)
Pre-eclampsia	29 (0.8)	5 (1.2)	33 (1.4)
Preterm delivery (<37 weeks’ gestation)	179 (4.7)	12 (2.8)	286 (11.8)
Birth weight (kg)*	3.3 ± 0.441	3.2 ± 0.465	3.2 ± 0.5
Birth weight <2500 g*	103 (2.8)	23 (5.6)	100 (4.7)
Newborn sex male	1903 (50.0)	199 (46.9)	1281 (52.7)

Table showing the demographics of the INTERGROWTH-21st dataset used to train the model, the separate dataset used for internal validation and the INTERBIO-21st dataset used for external validation of the model.

*≥37 weeks gestation only.

Data are given as mean ± SD or *n* (%).

Maternal baseline characteristics were measured at <14 weeks’ gestation.

*BMI* Body Mass Index, *GA* Gestational Age.

### Performance of single ultrasound planes versus MultiPlane

**Fig. 1:**
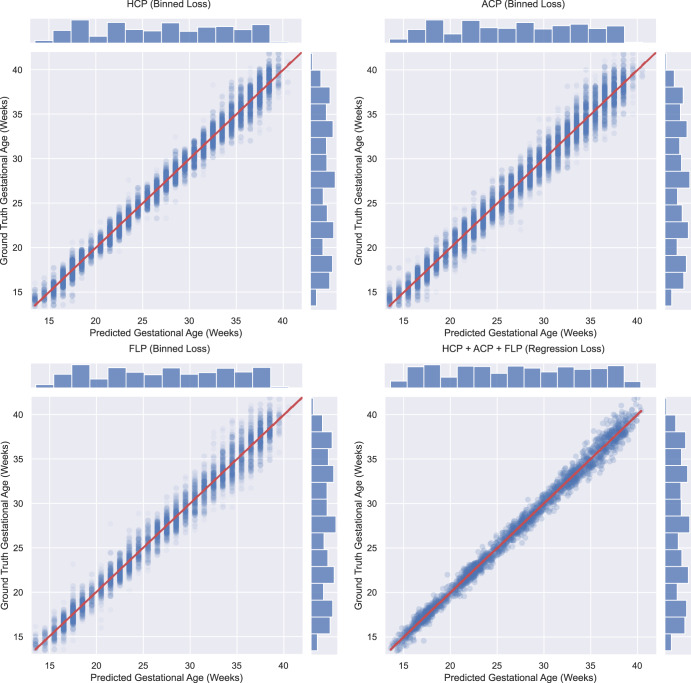
Performance per plane and MutliPlane on the INTERGROWTH-21st internal validation set. Composite figure showing scatter plot of the performance of the proposed single standard plane and MultiPlane models on the hold-out INTERGROWTH-21st test set with histograms showing the spread of planes across gestational ages. HCP Head Circumference Plane, ACP Abdominal Circumference Plane, FLP Femur Length Plane.

**Fig. 2:**
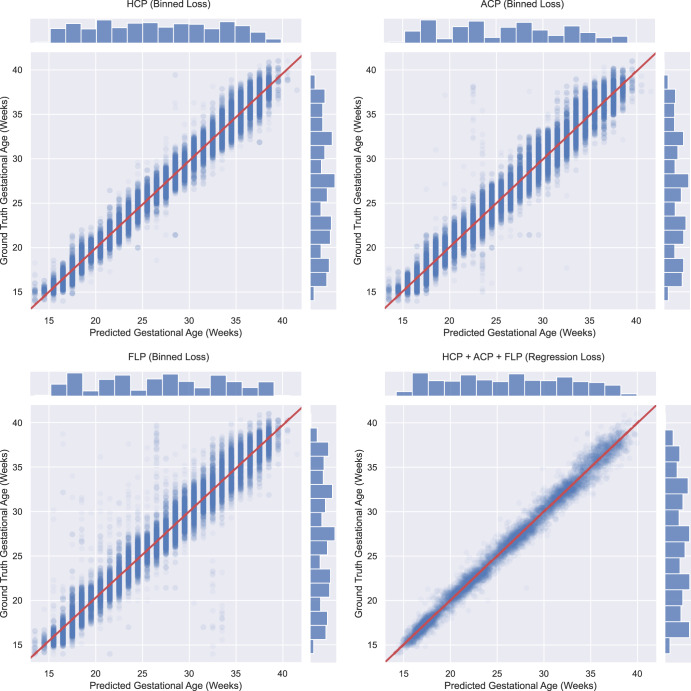
Performance per plane and MultiPlane on the INTERBIO-21st external validation set. Composite figure showing scatter plot of the performance of the proposed single standard plane and MultiPlane models on the INTERBIO-21st test set with histograms showing the spread of planes across gestational ages. HCP Head Circumference Plane, ACP Abdominal Circumference Plane, FLP Femur Length Plane.

Table 2 shows the performance of four different models: on the hold-out INTERGROWTH-21st Fetal Growth Longitudinal Study (FGLS) test set (Table 2a and Fig. 1) and the independent INTERBIO-21st Fetal Study dataset (Table 2b and Fig. 2) using a single standard plane HC only, AC only, FL only, and using all three standard planes (MultiPlane). Saliency maps demonstrate that the model used information for GA estimation using characteristics largely from within the fetal anatomy (see Supplementary Figs. 1, 2, and 3). MultiPlane outperforms HC only, AC only and FL only by 1, 2, and 3 days respectively for mean absolute error (MAE) across all GAs. We also analysed the performance of each model split by trimester, i.e., 18^+0^ to 27^+6^ weeks’ gestation (second trimester) and 28^+0^ to 42^+0^ weeks’ gestation (third trimester) with all models performing better in the second trimester.

**Table 2:** a Performance per plane in the INTERGROWTH-21st internal validation set.

			Algorithm performance			
			HC plane only	AC plane only	FL plane only	MultiPlane (HC+AC+FL)
INTERGROWTH-21^st^ (Test set)	GA 13^+0^–42^+0^ weeks (424 patients)	MAE (days)	±4.5	±5.8	±6.0	±3.5
		95% CI for MAE	(4.4–4.6)	(5.8–5.9)	(5.8–6.1)	(3.4–3.7)
		R^2^	0.99	0.98	0.98	0.99
		Estimated GA within ± 1 week of GA estimated by gold standard (%)	81.0%	70.4%	70.3%	90.7%
		Estimated GA within ± 2 weeks of GA estimated by gold standard (%)	97.3%	93.2%	93.3%	98.9%
	GA 18^+0^–27^+6^ weeks (419 patients)	MAE (days)	±3.9	±5.2	±5.3	±3.0
		95% CI for MAE	(3.8–4.0)	(5.1–5.3)	(5.2–5.5)	(2.9–3.2)
		R^2^	0.94	0.89	0.88	0.96
		Estimated GA within ± 1 week of GA estimated by gold standard (%)	86.9%	75.3%	74.8%	94.5%
		Estimated GA within ± 2 weeks of GA estimated by gold standard (%)	99.2%	96.1%	97.1%	99.7%
	GA 28^+0^–42^+0^ weeks (424 patients)	MAE (days)	±5.3	±6.9	±7.1	±4.3
		95% CI for MAE	(5.2–5.4)	(6.8–7.1)	(6.9–7.3)	(4.1–4.5)
		*R*^2^	0.91	0.85	0.85	0.94
		Estimated GA within ± 1 week of GA estimated by gold standard (%)	73.0%	61.2%	61.7%	85.6%
		Estimated GA within ± 2 weeks of GA estimated by gold standard (%)	95.1%	89.0%	88.5%	98.0%

b Performance per plane in the INTERBIO-21st external validation set						
			Algorithm performance			
			HC plane only	AC plane only	FL plane only	MultiPlane^a^ (HC+AC+FL)
INTERBIO-21^st^	GA 13^+0^–42^+0^ weeks (2443 Patients)	MAE (days)	±5.0	±6.1	±7.0	±4.1
		95% CI for MAE	(4.9–5.0)	(6.1–6.2)	(6.9–7.0)	(4.0–4.2)
		*R*^2^	0.98	0.97	0.95	0.99
		Estimated GA within ± 1 week of GA estimated by gold standard (%)	75.7%	66.7%	62.5%	85.1%
		Estimated GA within ± 2 weeks of GA estimated by gold standard (%)	96.3%	92.1%	89.7%	98.2%
	GA 18^+0^–27^+6^ weeks (1893 patients)	MAE (days)	±4.6	±5.8	±6.5	±3.7
		95% CI for MAE	(4.5–4.6)	(5.7–5.9)	(6.4–6.7)	(3.6–3.9)
		*R*^2^	0.91	0.86	0.79	0.94
		Estimated GA within ± 1 week of GA estimated by gold standard (%)	79.8%	68.6%	64.1%	88.1%
		Estimated GA within ± 2 weeks of GA estimated by gold standard (%)	97.5%	93.7%	91.5%	98.8%
	GA 28^+0^–42^+0^ weeks (1942 patients)	MAE (days)	±5.9	±7.3	±8.0	±5.0
		95% CI for MAE	(5.8–5.9)	(7.2–7.4)	(7.8–8.2)	(4.8–5.1)
		*R*^2^	0.86	0.77	0.69	0.90
		Estimated GA within ± 1 week of GA estimated by gold standard (%)	67.3%	58.6%	55.4%	78.1%
		Estimated GA within ± 2 weeks of GA estimated by gold standard (%)	93.9%	87.7%	85.8%	96.9%

Table showing the performance per plane (HC only, AC only, FL only, and MultiPlane) in the INTERGROWTH-21st test set (2a) and the INTERBIO-21st external validation set (2b).

*HC* Head Circumference, *AC* Abdominal Circumference, *FL* Femur Length, *MAE* Mean Absolute Error, *CI* Confidence Interval.

^a^As not all planes were available for all participants, MultiPlane is based on 2304 participants.

### Comparison with biometry-based methods for GA estimation

Further breakdown of MultiPlane’s performance is compared to existing, clinical, biometry-based methods for estimating GA that are currently in use, namely Hadlock^16^ and INTERGROWTH-21st^7^^,^ in Table 3. Here we see that biometry-based methods perform comparably to MultiPlane at earlier GAs, but that biometry-based GA estimation is less accurate beyond 32 weeks’ gestation with MultiPlane outperforming both biometry-based estimations^7,16^.

**Table 3:** Table showing comparison of MultiPlane performance to biometry-based methods of gestational age estimation throughout gestation.

		Biometry		MultiPlane
		Hadlock^16^ (BPD, HC, AC, FL)	INTERGROWTH-21st^7^ (HC & FL)	
INTERGROWTH-21st Internal Validation (424 patients)	14^+0^–19^+6^ weeks	2.6 (2.5 to 2.8)	2.1 (1.9 to 2.3)	2.6 (2.4 to 2.8)
	20^+0^–25^+6^ weeks	3.5 (3.2 to 3.7)	3.2 (2.9 to 3.4)	3.1 (2.8 to 3.3)
	26^+0^–31^+6^ weeks	4.8 (4.5 to 5.1)	4.8 (4.4 to 5.1)	3.3 (3.1 to 3.5)
	32^+0^–37^+6^ weeks	8.3 (7.8 to 8.9)	7.4 (6.8 to 8.0)	4.6 (4.3 to 4.9)
	≥38^+0^ weeks	15.5 (13.8 to 17.3)	11.7 (10.0 to 13.5)	5.4 (4.5 to 6.2)
	14^+0^–42^+0^ weeks	5.6 (5.3 to 5.8)	4.9 (4.6 to 5.1)	3.5 (3.4 to 3.7)
INTERBIO-21st External Validation (2304 patients)	14^+0^–19^+6^ weeks	2.3 (2.3 to 2.4)	2.5 (2.4 to 2.6)	2.9 (2.8 to 3.0)
	20^+0^–25^+6^ weeks	4.0 (3.9 to 4.1)	3.6 (3.5 to 3.7)	3.8 (3.6 to 3.9)
	26^+0^–31^+6^ weeks	5.4 (5.3 to 5.5)	5.5 (5.4 to 5.7)	4.1 (4.0 to 4.3)
	32^+0^–37^+6^ weeks	8.7 (8.4 to 8.9)	7.9 (7.7 to 8.2)	5.2 (5.0 to 5.4)
	≥38^+0^ weeks	13.9 (13.2 to 14.5)	10.6 (9.7 to 11.4)	7.5 (6.4 to 8.7)
	14^+0^–42^+0^ weeks	5.7 (5.6 to 5.7)	5.3 (5.2 to 5.4)	4.1 (4.0 to 4.2)
SGA cohort from INTERBIO-21st (326 patients)	14^+0^–19^+6^ weeks	1.8 (1.7 to 2.0)	2.0 (1.8 to 2.1)	2.7 (2.4 to 3.1)
	20^+0^–25^+6^ weeks	3.5 (3.3 to 3.7)	2.8 (2.6 to 3.0)	3.2 (2.7 to 3.6)
	26^+0^–31^+6^ weeks	4.7 (4.4 to 5.0)	4.4 (3.8 to 4.9)	3.6 (3.2 to 3.9)
	32^+0^–37^+6^ weeks	7.4 (6.9 to 8.0)	6.3 (5.8 to 6.9)	4.7 (4.2 to 5.1)
	≥38^+0^ weeks	15.6 (13.4 to 17.8)	11.8 (9.1 to 14.4)	4.6 (3.2 to 5.9)
	14^+0^–42^+0^ weeks	5.0 (4.7 to 5.2)	4.4 (4.1 to 4.7)	3.7 (3.5 to 3.9)
LGA cohort from INTERBIO-21st (201 patients)	14^+0^–19^+6^ weeks	2.3 (2.0 to 2.6)	2.5 (2.2 to 2.8)	3.3 (2.5 to 4.0)
	20^+0^–25^+6^ weeks	4.2 (3.8 to 4.6)	3.5 (3.1 to 3.8)	4.6 (3.6 to 5.5)
	26^+0^–31^+6^ weeks	5.9 (5.4 to 6.5)	4.8 (4.2 to 5.3)	5.1 (4.3 to 5.8)
	32^+0^–37^+6^ weeks	10.6 (9.7 to 11.5)	7.9 (6.8 to 9.0)	5.3 (4.5 to 6.1)
	≥38^+0^ weeks	15.3 (9.2 to 21.5)	10.2 (5.9 to 14.5)	Insufficient data
	14^+0^–42^+0^ weeks	6.2 (5.8 to 6.6)	4.9 (4.5 to 5.3)	4.7 (4.3 to 5.1)

Table showing performance of MultiPlane and current biometry methods used for estimating gestational age throughout gestation. Figures shown are mean absolute error (MAE) in days with the 95% confidence interval for the MAE in parenthesis. *BPD* Biparietal Diameter, *HC* Head Circumference, *AC* Abdominal Circumference, *FL* Femur Length, *SGA* Small for Gestational Age, *LGA* Large for Gestational Age.

### MultiPlane performance

In internal validation (INTERGROWTH-21st dataset), for all GAs from 13^+0^ to 42^+0^ weeks, we achieved a MAE in GA estimation of 3.5 days for the MultiPlane model, with 90.7% within ±7 days of the gold standard. The proportion estimated within ±7 days of the gold standard was 94.5% in the second trimester and 85.6% in the third trimester. For GAs between 14^+0^ and 27^+6^ weeks, arguably the more important GA window clinically in LMICs, the MAE was 3.0 days and 94.5% were correctly estimated within ±7 days of the gold standard (Table 2a).

In the external validation dataset (INTERBIO-21st), the MultiPlane model achieved a MAE of 4.1 days across the entire GA range (13^+0^ to 42^+0^ weeks), with 85.1% estimated within ±7 days of the gold standard. For GAs between 14^+0^ and 27^+6^ weeks, there was a MAE of 3.7 days with 88.1% estimated within ±7 days from the gold standard (Table 2b). Thus, there was no significant MAE loss when comparing the INTERBIO-21st dataset to the INTERGROWTH-21st test set despite the higher rates in the INTERBIO-21st study of maternal and perinatal complications, including SGA, associated with a much less healthy cohort of mothers (Table 2a, b).

**Fig. 3:**
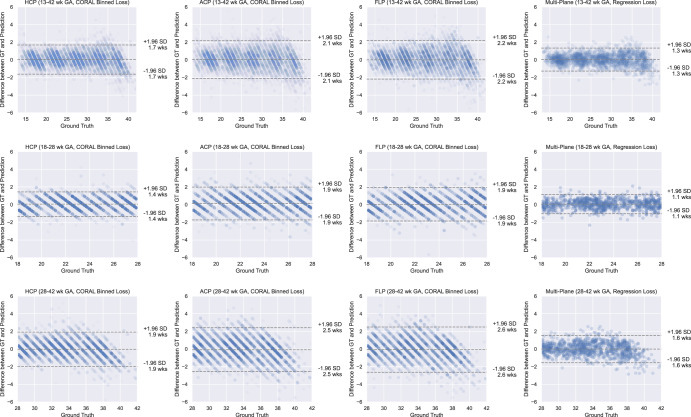
Modified Bland-Altman plots showing per plane performance on the INTERGROWTH-21st internal validation set. Modified Bland-Altman plots of the performance of the gestational age (GA) estimation models on the INTERGROWTH-21st dataset. The striations visible on the data points for the single standard plane models are due to the CORAL binning process employed during training, which is removed for the final MultiPlane regression loss. Due to the variation in the number of data points per figure, the transparency of each scatter point was normalised so that the overall appearance is normalised. HCP Head Circumference Plane; GA Gestational Age; CORAL Consistent Ordinal RAnk Logits; GT Ground Truth; SD Standard Deviation; ACP Abdominal Circumference Plane; FLP Femur Length Plane.

Modified Bland–Altman plots are shown in Fig. 3 for the GA estimation models, with ground truth plotted on the *x*-axis instead of the mean of the measures. As expected, the binning procedure during the initial pre-training step led to stratification of the predicted GAs. Furthermore, using all three standard planes of a single fetus during inference reduced the standard deviation (SD) of difference across all GAs. The plot also shows that there was no systematic over- or under-estimation of GA across the evaluated GA range on the overall dataset.

### Performance in SGA and LGA fetuses

**Fig. 4:**
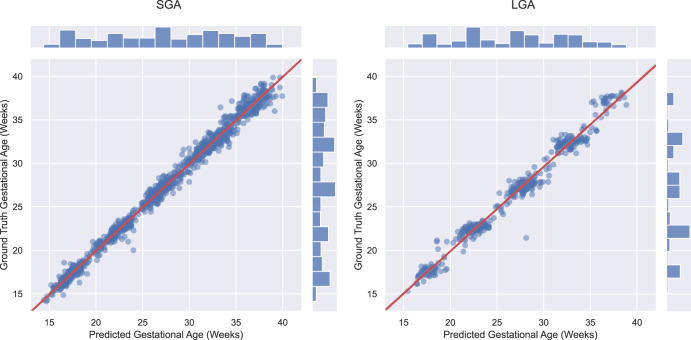
MultiPlane performance in small and large for gestational age fetuses. Gestational age (GA) estimation results in pregnancies resulting in small for gestational age (SGA) or large for gestational age (LGA) newborns, defined by a birth weight >90th centile (LGA) or <10th centile (SGA) according to the INTERGROWTH-21st international standard^30^.

In a sub-analysis, we investigated model accuracy for SGA (*n* = 326) and LGA (*n* = 201) newborns in the INTERBIO-21st study. The best performing MultiPlane algorithm predicted GA across the second and third trimesters to 3.7 (95% Confidence Interval (CI); 3.5 to 3.9) and 4.7 (95% CI; 4.3 to 5.1) days for SGA and LGA newborns, respectively (Table 3 and Fig. 4). We compared this performance to biometry estimates for these fetuses and found that biometry-based estimates were more prone to error than MultiPlane beyond 32^+0^ weeks’ gestation (Table 3) with a MAE of 7.4 and 10.6 days for SGA and LGA using the Hadlock^16^ formula compared with 4.7 and 5.3 days for MultiPlane, respectively.

### Review of outliers

An expert sonologist (EB) qualitatively reviewed images that were outliers (estimation error >14 days). No obvious systematic clinical or image features resulting in inaccurate estimation by the models were evident, although we did identify a pregnancy affected by achondroplasia (only apparent after 24 weeks’ gestation). The model performed well with a 0.3-week estimation error until the scan at 36 weeks; at this point the error was greater than 3 weeks.

### Performance of MultiPlane per site

Subanalysis of the MultiPlane performance by site participating in the external validation INTERBIO-21st is shown in Table 4, demonstrating high performance throughout, and all differences between sites within 0.5 days from the pooled MAE, which is clinically insignificant.

**Table 4:** Performance of gestational age assessment using MultiPlane per study site.

	Study site						Overall (2304 patients)
	A (287 patients)	B (492 patients)	C (565 patients)	D (142 patients)	E (462 patients)	F (392 patients)	
MAE (days)	3.6	4.2	3.8	3.8	4.6	4.3	4.1
95% CI MAE (days)	(3.4 to 3.8)	(4.0 to 4.4)	(3.6 to 3.9)	(3.4 to 4.2)	(4.4 to 4.8)	(4.0 to 4.6)	(4.0 to 4.2)

Table showing the performance of MultiPlane for individual study sites included in the INTERBIO-21st study throughout the second and third trimester, coded A–E. *MAE* Mean Absolute Error, *CI* Confidence Interval, *GA* Gestational Age.

## Discussion

In this study, we have developed and applied machine learning models that analyse fetal ultrasound images to estimate GA, which have been validated on a separate large dataset that was unseen during model training. The images were acquired prospectively by trained sonographers in the context of two independent research studies with the same protocol and ultrasound machines. All included pregnancies were dated using: (a) CRL (INTERBIO-21st) or (b) the certain LMP if it was ≤7 days of the CRL dating (INTERGROWTH-21st), which was used as the ground truth to train the model and evaluate the results. Comparing our results to current clinical practice for dating pregnancies in the second and third trimesters based on fetal biometry^7,16^, we report greatly improved GA estimation accuracy, especially in late pregnancy. This is highly relevant clinically as late pregnancy assessment of GA has long been problematic due to large variability in biological size, greater absolute scanning error, and a higher incidence of SGA and LGA.

There are several important strengths to this work. Our training dataset, encompassing the second and third trimesters, was large and acquired under standardised conditions from eight study sites on five continents. It represents a well-phenotyped cohort of healthy fetuses with satisfactory growth and neurodevelopment up to 2 years of age^17^. Ground truth was rigorously established based on certain LMP and regular 24–32 day menstrual cycles, corroborated by CRL measurement at <14^+0^ weeks’ gestation. Although originally developed on a low-risk population we have validated these GA estimation models on a separate dataset from a cohort with broader inclusion criteria reflecting real-world scenarios. This has allowed us to demonstrate that our model works in a population comprising high and low-risk pregnancies as well as in different geographical settings. Through sub-group analysis, we have demonstrated that MultiPlane is more resistant to errors in GA estimation for SGA and LGA fetuses beyond 32^+0^ weeks’ gestation as it was developed without information relating to scale and it relies solely on the appearance of the three standard ultrasound planes. Ground truth in the INTERBIO-21st study was established by measuring CRL < 14^+0^ weeks’ gestation in every case. We found that GA estimation performance on this second dataset was comparable to the hold-out testing set of the original dataset. Crucially, we did not utilise any biometric information in model training or testing. Rather, our solution, which works in real-time, uses and combines image analysis of multiple anatomical planes that are routinely acquired as part of standard clinical scans, allowing a single GA estimate at any time point of pregnancy. This was found to be more accurate than existing methods.

For context, we have also compared our work to current methods of GA estimation, namely clinical biometry-based methods (Hadlock^16^ and INTERGROWTH-21st^7^^,^), and showed that our MultiPlane model is the most accurate for late third-trimester GA estimation. We have also compared the MultiPlane model to a machine-learning model that uses only fetal head ultrasound images for GA estimation^14^. Our MultiPlane model outperforms this, as well as an additional method that takes as input fetal biometry during inference in the third trimester (7.1 and 5.5 days respectively vs. 4.3 days).

The generalisability of the MultiPlane model for images acquired with other ultrasound machines requires future assessment as our data were acquired using the same type of ultrasound machine across all eight study sites. It is possible that our method would not perform as accurately “as-is” when presented with standard planes acquired using other ultrasound machines. However, we believe that we have for the first time established the principle that GA can accurately be assessed based on Multiplane image characteristics, and emerging techniques to overcome the domain shift in image characteristics could be used to fine-tune the machine-learning models. The requirement to retrain models on data from a new domain to achieve the highest possible diagnostic accuracy is a recognised limitation of current machine learning modelling techniques, rather than a limitation specific to our application. It should be said that, while our models were built on large datasets, relatively small amounts of data from a new domain would be expected to allow a model to adapt to images from a different ultrasound machine. Fetal abnormalities were not excluded from our dataset. However, one of the scans MultiPlane estimated to be >3 weeks from the ground truth was a case with achondroplasia; hence, further analysis of the performance in fetal abnormalities is warranted to understand how the model is affected. We have demonstrated that our model works well in cases of SGA and LGA; thus, when a discrepancy between the MultiPlane GA estimate and biometry suggests growth restriction, a repeat scan time to monitor fetal growth would be a reasonable precaution.

One challenge to implementation is that the MultiPlane model requires standard anatomical planes as input. It is, therefore, dependent on the acquisition of these planes by healthcare providers trained in ultrasonography. In practice, such planes are part of standard ultrasound examination to assess fetal growth and wellbeing, and with appropriate training can be acquired by local health workers in an LMIC setting^18^. The availability of these skilled professionals and the possibility of human error during acquisition are potential limitations to implementation, which may be overcome by integration with machine-learning-based methods for automated standard plane detection^19,20^. Other barriers to routine ultrasound in LMICs such as cost, maintenance and repair remain important considerations for implementation, and successful integration of these automated algorithms into low-cost point of care devices should be considered.

We have demonstrated that MultiPlane improves on current biometry-based methods of GA estimation in the second and third trimesters, even in cases of growth aberration. It, therefore, has the potential to improve the care of women and babies, especially in LMICs where GA is unknown in half of all pregnancies^1^.

## Methods

The Biometry Automation in OBstetrics And Beyond (BAOBAB) Study aims to overcome roadblocks to effective pregnancy ultrasound in LMICs by bringing together engineers, clinician-scientists and healthcare providers, and by learning from existing studies wherever possible. In this study our aim was to establish whether machine learning of image-based appearance of standard biometric planes is associated with GA; and whether this is sufficiently discriminatory to be of clinical utility. The ground truth for GA estimation was based on first trimester CRL measurements or LMP if this was within ≤7 days of the CRL (see below for details).

### Fetal ultrasound datasets

We developed a machine learning model based on ultrasound images from two independent datasets. Ultrasound images from the Fetal Growth Longitudinal Study (FGLS) of the INTERGROWTH-21st^21^, Project were used to train, validate and test (internally validate) the model. External validation was then performed on ultrasound images from the INTERBIO-21st Fetal Study^22^ which were unseen by the model during the development phase. During internal validation (using INTERGROWTH-21st data) and external validation (using INTERBIO-21st data) the model was blinded to the ground truth.

### Training and internal validation

INTERGROWTH-21st was a multicentre, multiethnic, population-based project, conducted between 2009 and 2014 in eight countries. The primary aim was to study growth, health, nutrition, and neurodevelopment from less than 14 weeks’ gestation to 2 years of age. Details of the study have been described elsewhere^23–25^. In brief, all institutions providing obstetric care in eight geographically diverse regions in Brazil, China, India, Italy, Kenya, Oman, UK, and USA were chosen as study sites. From these, healthy women with a naturally conceived, singleton pregnancy who were at low risk of adverse maternal and perinatal outcomes were prospectively enroled into FGLS, one of the main components of INTERGROWTH-21st. GA was estimated from the LMP provided that: (a) the date was certain; (b) the woman had a regular 24–32 day menstrual cycle; (c) she had not been using hormonal contraception or breastfeeding in the preceding 2 months, and (d) any discrepancy between the GAs based on LMP and CRL, measured by ultrasound at 9^+0^ to 13^+6^ weeks from the LMP was ≤7 days^26^.

Trained, dedicated research sonographers performed ultrasound scans every 5^±1^ weeks using identical equipment at all sites (Philips HD9 [Philips Ultrasound, Bothell, WA, USA] with curvilinear abdominal transducers C5–2, C6-3, V7-3). We used stored images of the three standard anatomical planes: (a) fetal head in the axial view at the level of the thalami, as required for measurement of the HC; (b) abdomen in an axial view at the level of measurement of the AC, and (c) femur in the longitudinal view used for measuring FL. The detailed measurement protocol, training, standardisation, and quality-control methods, including quality scoring of images, used across all study sites are described in detail elsewhere^25,27,28^ and all documentation, protocols, data collection forms, and electronic transfer strategies are freely available on the INTERGROWTH-21st website.

### External validation

The INTERBIO-21st Fetal Study was conducted, between 2012 and 2019, at six sites in Pelotas (Brazil), Nairobi (Kenya), Karachi (Pakistan), Soweto (South Africa), Mae Sot (Thailand) and Oxford (UK), all sites were urban, except Mae Sot which was a rural site^29^. All aspects of the study, including the ultrasound protocol, were identical to FGLS except that GA was estimated by CRL measurement at <14 weeks’ gestation (as a certain LMP would not be expected in a large proportion of this high-risk cohort owing to maternal conditions, poor nutrition, anaemia etc); thus, any woman with a singleton pregnancy was eligible. Hence, the population was more heterogenous and at higher risk of fetal growth impairment because of exposures such as HIV, malaria, and malnutrition, adding external validity to the automated GA estimation model.

FGLS provided 293,811 images from 4233 pregnancies which were randomly split in the following manner on a per fetus basis: 75% for model training (219,974 images), 15% for validation (44,173 images) and 10% for testing (29,664 images). The validity of the GA estimation model was then tested on 94,832 images from 2443 pregnancies in the INTERBIO-21st Fetal Study. Due to the longitudinal nature of both studies, the data were selected with evenly distributed GAs across the second and third trimesters.

In a sub-analysis, we further validated the automated GA estimation model in SGA and LGA pregnancies, as these pose a particular challenge in GA estimation using methods based on biometry. These were identified by birth weight for GA and sex below the 10th or above the 90th centiles, respectively^30^. Further analysis comparing MultiPlane to biometry-based estimates (Hadlock^16^ and INTERGROWTH-21st^7^^,^) was also performed for all pregnancies in the INTERGROWTH-21st test set and INTERBIO-21st. Finally, we performed subanalysis of MultiPlane’s performance across the participating sites in the INTERBIO-21st dataset.

### Image pre-processing and augmentation

It is important to note that all measurement or scale information was removed from ultrasound images. INTERGROWTH-21st and INTERBIO-21st images included sonographer markings such as measurement calipers and other text annotations. The locations of these artefacts were found using image cross-correlation-based template matching, and anomalous pixels were smoothed with bilinear intensity interpolation. Images were then paired with their respective ground truth GAs, as described above. Crucially, at no point was the GA estimation algorithm provided with any biometric information, image calibration markings or pixel size, including the centimetre “rule” within ultrasound images; it was therefore entirely reliant on the appearance of the images for GA estimation.

Images were first downsampled to 224×224 pixels using bilinear interpolation. An experienced sonographer (EB) then manually validated a subset of the resized images (*n* = 100 per standard plane) to check that the resizing and interpolation procedure using this subset did not invalidate the planes. Images were then intensity normalised to have zero mean and unary SD per individual image to aid in convolutional neural network (CNN) model convergence.

### Algorithm design and development

The CNN model architecture used was based on the ResNet-50 network^31^ as a backbone, an architecture routinely used in natural image analysis. The CNN architecture consists of modularised convolutional layers characterised with skip connections between modules. We modified the standard CNN architecture with two major components: (i) Consistent Ordinal RAnk Logits (CORAL) for Classification Loss^32^ during single standard plane pre-training and (ii) plane-specific pre-trained models for automated feature extraction.

**Fig. 5:**
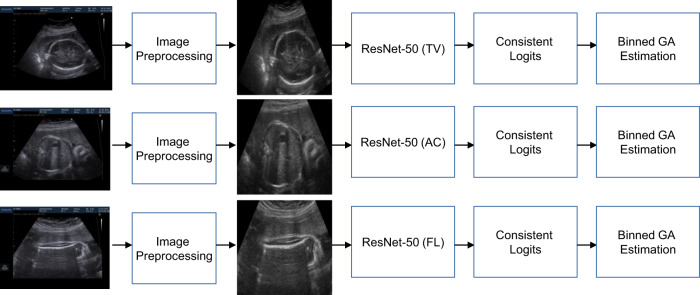
Training process for single plane estimation of gestational age. Schematic of training process for single ultrasound standard plane-based gestational age (GA) estimation using Consistent Rank Logit Loss. HC Head Circumference; GA Gestational Age; AC Abdominal Circumference; FL Femur Length.

Neural networks perform worse on regression tasks when trained from scratch with a regression-based loss, such as the MAE^33^, because large outlier predictions can lead to drastic gradient updates during back-propagation, which results in network instability during the initial stages of training. Consequently, naively treating GA estimation as a regression problem may reduce final accuracy. So, “binned” classification loss is commonly used when training neural networks for regression, where the final target variable is binned before classification loss is applied. However, ordinal information is lost using this method. Therefore, we used CORAL to train the network as this retains the ordinal information between each bin^32^ but has the stability of binned classification-based training for neural networks. In our case, GA data were binned into integers per week during the training of single-plane networks before being optimised with CORAL for Classification Loss (Fig. 5).

**Fig. 6:**
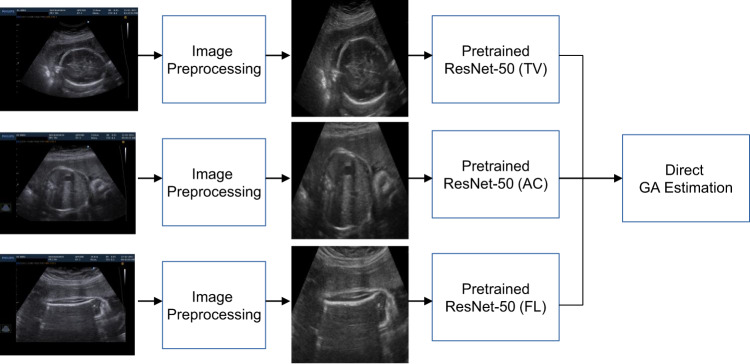
Training process for MultiPlane model for gestational age estimation. Schematic of training process for multiple ultrasound standard plane-based gestational age (GA) estimation. Pre-trained models are based on single standard plane images trained with Consistent Rank Logit Loss. The final multiple standard plane model is trained with L1-loss. HC Head Circumference; AC Abdominal Circumference; FL Femur Length; GA Gestational Age.

We hypothesised that there was additional information in each standard plane that could be independently used for more accurate GA estimation. The Multiplane model was based on a concatenation of the final layers of the pre-trained single-plane models with pre-trained weights, and subsequently fine-tuned using multiple planes (HC, AC, FL) from the same fetus in an additional training loop. We used L1 regularisation to minimise the loss gradients of outliers during the training process, and found that this provided a good final performance (see Fig. 6).

All CNN architectures were trained and tested on a 75, 15, and 10% split of training, validation and test, respectively (on a per-fetus basis) in the INTERGROWTH-21st dataset, ensuring no overlap between groups. As recommended^34^, the INTERBIO-21^st^ dataset was used solely for final model testing, and no models were exposed to any INTERBIO-21st images during the training process, allowing the INTERBIO-21^st^ dataset to be used to study model generalisability.

### Implementation details

All models were implemented using PyTorch (1.1.0). Model training was performed on an NVIDIA Tesla V100, and the optimised convergence of each GA estimation model was found in approximately 48 h per single standard plane model. The convergence and fine-tuning of a multiple standard plane model using the single standard plane models as pre-trained weights took a further 12 h, with images augmented in a process described elsewhere^19^. However, after training and convergence, inference with new images could be performed on an average of 39 (95% CI; 35 to 43) frames/s, which is fast enough for inference during real-time ultrasound scanning.

### Inclusion and ethics

The INTERGROWTH-21st Project protocol and amendment extension to INTERBIO-21st were approved by the Oxfordshire Research Ethics Committee C (reference: 08/H0606/139); all the pregnant women enroled gave informed written consent.

### Reporting summary

Further information on research design is available in the Nature Research Reporting Summary linked to this article.

## Supplementary information

Supplementary figures. Machine Learning for Accurate Gestational Age Estimation. Lee, Bradburn et al.pdf

REPORTING SUMMARY

## Data Availability

All documentation, protocols, data collection forms, and clinical tools are freely available on the INTERGROWTH-21st website (https://intergrowth21.tghn.org/). Ultrasound images cannot be made available due to ethical approval restrictions.

## Code availability

10.5281/zenodo.7551882; codebase is open source and published under the MIT License. All trained models and configurations have however been removed.
