# Supplementary material for: Machine learning for accurate estimation of fetal gestational age based on ultrasound images
Source: NPJ Digit Med. 2023 Mar 9;6:36. doi: 10.1038/s41746-023-00774-2 (PMC9998590; doi:10.1038/s41746-023-00774-2)
Supplement: Supplementary file 1 — Supplementary figures. Machine Learning for Accurate Gestational Age Estimation. Lee, Bradburn et al.pdf [file 41746_2023_774_MOESM1_ESM.pdf]

# Supplementary Figures and Acknowledgements: Machine Learning for Accurate Estimation of Fetal Gestational Age Based on Ultrasound Images

Lok Hin Lee[1]*, Elizabeth Bradburn[2]*, Rachel Craik[2], Mohammad Yaqub[3], Shane A Norris[4], Leila Cheikh Ismail[5], Eric O Ohuma[2,6], Fernando C Barros[7,8], Ann Lambert[2], Maria Carvalho[9], Yasmin A Jaffer[10], Michael Gravett[11], Manorama Purwar[12], Qingqing Wu[13], Enrico Bertino[14], Shama Munim[15], Aung Myat Min[16], Zulfiqar Bhutta[15,17], Jose Villar[2,18], Stephen H Kennedy[2,18], J Alison Noble[1] ** and Aris T Papageorghiou[2,18]**

*Joint first authors ** Joint senior authors

1. Institute of Biomedical Engineering, Department of Engineering Science, University of Oxford, Oxford, UK
2. Nuffield Department of Women's & Reproductive Health, University of Oxford, Oxford, UK
3. Intelligent Ultrasound Ltd, Hodge House, Cardiff, CF10 1DY
4. South African Medical Research Council Developmental Pathways for Health Research Unit, Department of Paediatrics & Child Health, University of the Witwatersrand, Johannesburg, South Africa
5. College of Health Sciences, University of Sharjah, University City, United Arab Emirates
6. Maternal, Adolescent, Reproductive & Child Health (MARCH) Centre, London School of Hygiene & Tropical Medicine, London, UK
7. Programa de Pós-Graduação em Epidemiologia, Universidade Federal de Pelotas, Pelotas, Brazil
8. Programa de Pós-Graduação em Saúde e Comportamento, Universidade Católica de Pelotas, Pelotas, Brazil
9. Faculty of Health Sciences, Aga Khan University, Nairobi, Kenya
10. Department of Family & Community Health, Ministry of Health, Muscat, Oman
11. Departments of Obstetrics and Gynecology and of Global Health, University of Washington, Seattle, WA, USA
12. Nagpur INTERGROWTH-21st Research Centre, Ketkar Hospital, Nagpur, India
13. School of Public Health, Peking University, Beijing, China
14. Dipartimento di Scienze Pediatriche e dell' Adolescenza, Struttura Complessa Direzione Universitaria Neonatologia, Università di Torino, Torino, Italy
15. Department of Obstetrics & Gynaecology, Division of Women & Child Health, Aga Khan University, Karachi, Pakistan
16. Shoklo Malaria Research Unit, Mahidol-Oxford Tropical Medicine Research Unit, Faculty of Tropical Medicine, Mahidol University, Mae Sot, Tak, Thailand
17. Center for Global Child Health, Hospital for Sick Children, Toronto, Canada
18. Oxford Maternal & Perinatal Health Institute, Green Templeton College, University of Oxford, Oxford, UK

**Supplementary Figure 1: Saliency maps for fetuses <23$^{+0}$ weeks**

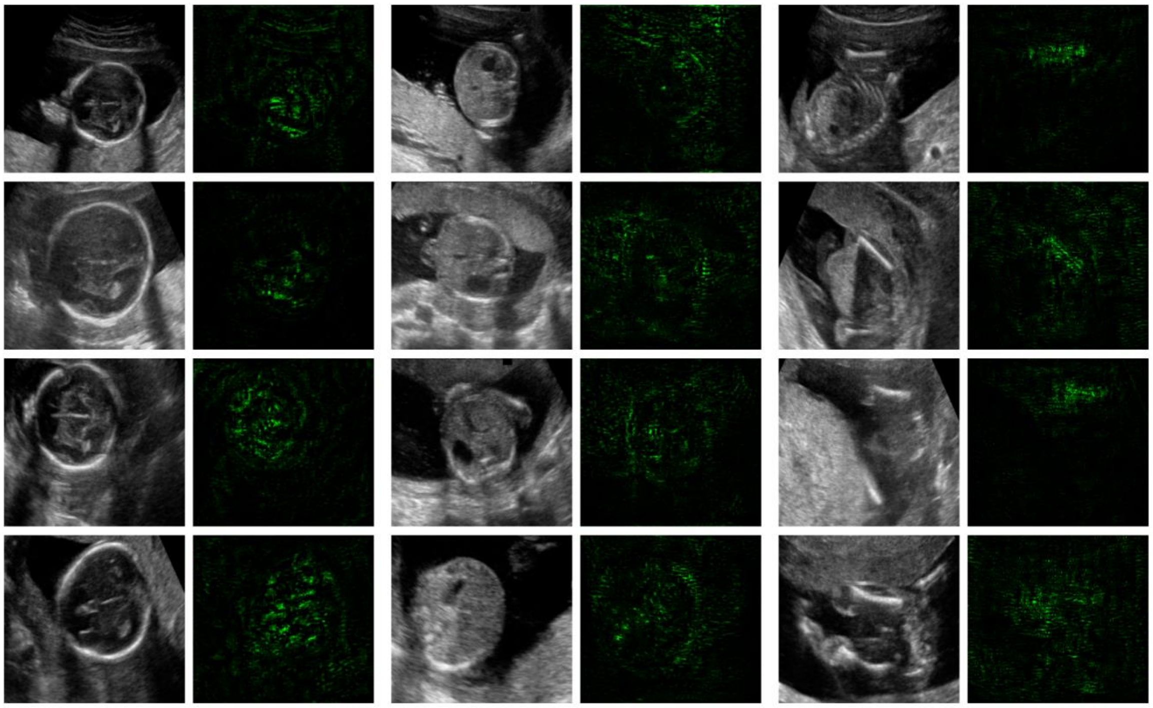

Saliency maps for fetuses where gestational age was less than 23$^{+0}$ weeks. The head circumference, abdominal circumference and femur length planes are shown in columns 1, 2 and 3 respectively. The corresponding saliency map is shown adjacent to the fetal plane image to which it relates. The green indicates the degree of attribution to each individual pixel used for the model's assessment of gestational age. Saliency maps were generated using Integrated Gradients[1]. The images are taken from a random sample of all included fetuses.

**Supplementary Figure 2**: **Saliency maps for fetuses ≥$23^{+0}$ weeks and ≤$30^{+6}$ weeks**

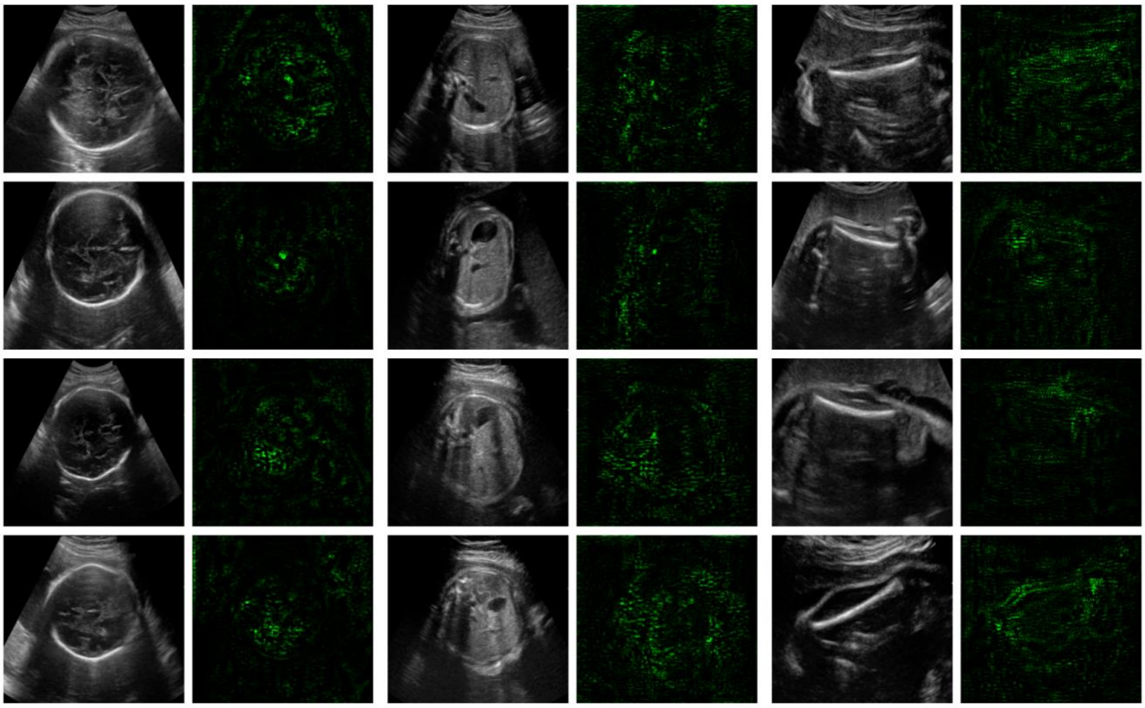

Saliency maps for fetuses where gestational age was between $23^{+0}$ - $30^{+6}$ weeks. The head circumference, abdominal circumference and femur length planes are shown in columns 1, 2 and 3 respectively. The corresponding saliency map is shown adjacent to the fetal plane image to which it relates. The green indicates the degree of attribution to each individual pixel used for the model's assessment of gestational age. Saliency maps were generated using Integrated Gradients[1]. The images are taken from a random sample of all included fetuses.

**Supplementary Figure 3: Saliency maps for fetuses ≥$31^{+0}$ weeks**

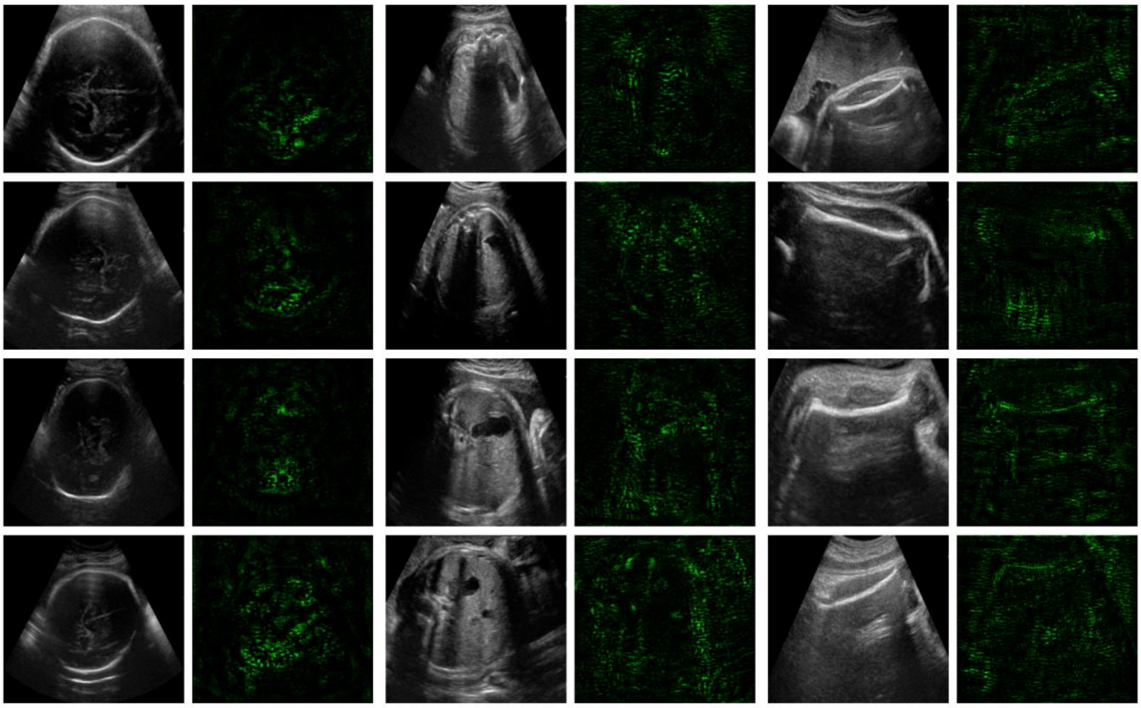

Saliency maps for fetuses where gestational age was more than $31^{+0}$ weeks. The head circumference, abdominal circumference and femur length planes are shown in columns 1, 2 and 3 respectively. The corresponding saliency map is shown adjacent to the fetal plane image to which it relates. The green indicates the degree of attribution to each individual pixel used for the model's assessment of gestational age. Saliency maps were generated using Integrated Gradients[1]. The images are taken from a random sample of all included fetuses.

[1]Sundararajan, M., Taly, A., Yan, Q. Axiomatic attribution for deep networks. International conference on machine learning (2017).

## Acknowledgements:

Full acknowledgement of all those who contributed to the development of the INTERGROWTH-21st Project protocol appears at www.intergrowth21.org.uk. Full acknowledgement for all those who contributed to the development of the Project protocol appears at www.interbio21.org.uk

**Members of the International Fetal and Newborn Growth Consortium for the 21st Century (INTERGROWTH-21st) and its committees**

*Scientific Advisory Committee*

M Katz (Chair from January 2011), MK Bhan, C Garza, S Zaidi, A Langer, PM Rothwell (from February 2011), Sir D Weatherall (Chair until December 2010).

*Steering Committee*

ZA Bhutta (Chair), J Villar (Principal Investigator), S Kennedy (Project Director), DG Altman (deceased 2018), FC Barros, E Bertino, F Burton, M Carvalho, L Cheikh Ismail, WC Chumlea, MG Gravett, YA Jaffer, A Lambert, P Lumbiganon, JA Noble, RY Pang, AT Papageorghiou, M Purwar, J Rivera, C Victora.

*Executive Committee*

J Villar (Chair), DG Altman (deceased 2018), ZA Bhutta, L Cheikh Ismail, S Kennedy, A Lambert, JA Noble, AT Papageorghiou.

*Project Coordinating Unit*

J Villar (Head), S Kennedy, L Cheikh Ismail, A Lambert, AT Papageorghiou, M Shorten, L Hoch (until May 2011), HE Knight (until August 2011), EO Ohuma (from September 2010), C Cosgrove (from July 2011), I Blakey (from March 2011), D Bishop (from February 2014).

*Data Analysis Group*

DG Altman (Head, deceased 2018), EO Ohuma, J Villar.

*Data Management Group*

DG Altman (Head, deceased 2018), F Roseman, N Kunnawar, SH Gu, JH Wang, MH Wu, M Domingues, P Gilli, L Juodvirsiene, L Hoch (until May 2011), N Musee (until June 2011), H Al-Jabri (until October 2010), S Waller (until June 2011), C Cosgrove (from July 2011), D

Muninzwa (from October 2011), EO Ohuma (from September 2010), D Yellappan (from November 2010), A Carter (from July 2011), D Reade (from June 2012), R Miller (from June 2012).

*Ultrasound Group*

AT Papageorghiou (Head), L Salomon (Senior external advisor), A Leston, A Mitidieri, F Al-Aamri, W Paulsene, J Sande, WKS Al-Zadjali, C Batiuk, S Bornemeier, M Carvalho, M Dighe, P Gaglioti, N Jacinta, S Jaiswal, JA Noble, K Oas, M Oberto, E Olearo, MG Owende, J Shah, S Sohoni, T Todros, M Venkataraman, S Vinayak, L Wang, D Wilson, QQ Wu, S Zaidi, Y Zhang, P Chamberlain (until September 2012), D Danelon (until July 2010), I Sarris (until June 2010), J Dhami (until July 2011), C Ioannou (until February 2012), CL Knight (from October 2010), R Napolitano (from July 2011), S Wanyonyi (from May 2012), C Pace (from January 2011), V Mkrtychyan (from June 2012).

*Anthropometry Group*

L Cheikh Ismail (Head), WC Chumlea (Senior external advisor), F Al-Habsi, ZA Bhutta, A Carter, M Alija, JM Jimenez-Bustos, J Kizidio, F Puglia, N Kunnawar, H Liu, S Lloyd, D Mota, R Ochieng, C Rossi, M Sanchez Luna, YJ Shen, HE Knight (until August 2011), DA Rocco (from June 2012), IO Frederick (from June 2012).

*Neonatal Group*

ZA Bhutta (Head), E Albernaz, M Batra, BA Bhat, E Bertino, P Di Nicola, F Giuliani, I Rovelli, K McCormick, R Ochieng, RY Pang, V Paul, V Rajan, A Wilkinson, A Varalda (from September 2012).

*Infant Development Group*

M Fernandes (Head), A Stein, F Guiliani, K Wulff, AA Abubakar, C Newton.

*Environmental Health Group*

B Eskenazi (Head), LA Corra, H Dolk, J Golding, A Matijasevich, T de Wet, JJ Zhang, A Bradman, D Finkton, O Burnham, F Farhi.

*Participating countries and local investigators*

*Brazil*: FC Barros (Principal Investigator), M Domingues, S Fonseca, A Leston, A Mitidieri, D Mota, IK Sclowitz, MF da Silveira.

*China*: RY Pang (Principal Investigator), YP He, Y Pan, YJ Shen, MH Wu, QQ Wu, JH Wang, Y Yuan, Y Zhang.

*India*: M Purwar (Principal Investigator), A Choudhary, S Choudhary, S Deshmukh, D Dongaonkar, M Ketkar, V Khedikar, N Kunnawar, C Mahorkar, I Mulik, K Saboo, C Shembekar, A Singh, V Taori, K Tayade, A Somani.

*Italy*: E Bertino (Principal Investigator), P Di Nicola, M Frigerio, G Gilli, P Gilli, M Giolito, F Giuliani, M Oberto, L Occhi, C Rossi, I Rovelli, F Signorile, T Todros.

*Kenya*: W Stones and M Carvalho (Co-Principal Investigators), J Kizidio, R Ochieng, J Shah, , S Vinayak, N Musee (until June 2011), C Kisiang'ani (until July 2011), D Muninzwa (from August 2011).

*Oman*: YA Jaffer (Principal Investigator), J Al-Abri, J Al-Abduwani, FM Al-Habsi, H Al-Lawatiya, B Al-Rashidiya, WKS Al-Zadjali, FR Juangco, M Venkataraman, H Al-Jabri (until October 2010), D Yellappan (from November 2010).

*UK*: SH Kennedy (Principal Investigator), L Cheikh Ismail, AT Papageorghiou, F Roseman, A Lambert, EO Ohuma, S Lloyd, R Napolitano (from July 2011), C Ioannou (until February 2012), I Sarris (until June 2010).

*USA*: MG Gravett (Principal Investigator), C Batiuk, M Batra, S Bornemeier, M Dighe, K Oas, W Paulsene, D Wilson, IO Frederick, HF Andersen, SE Abbott, AA Carter, H Algren, DA Rocco, TK Sorensen, D Enquobahrie, S Waller (until June 2011).

**Members of the INTERBIO-21$^{st}$ Consortium and its committees**

*Scientific Advisory Committee*

M Katz (Chair), MK Bhan, C Garza, A Langer, PM Rothwell, S Zaidi.

*Steering Committee*

R Uauy (Chair), SH Kennedy (Co-Principal Investigator), J Villar (Co-Principal Investigator), DG Altman, FC Barros, JA Berkley, F Burton, M Carvalho, L Cheikh Ismail, WC Chumlea, A Lambert, S Munim, S Norris, F Nosten, AT Papageorghiou, C Victora.

*Executive Committee*

J Villar (Chair), DG Altman (died 2018), L Cheikh Ismail, R Craik, SH Kennedy, A Lambert, AT Papageorghiou, R Uauy.

*Study Coordinating Unit*

J Villar (Head), S Ash, R Craik, L Cheikh Ismail, SH Kennedy, A Lambert, AT Papageorghiou, M Shorten.

*Data Analysis Group*

DG Altman (Head), EO Ohuma, AT Papageorghiou, E Staines Urias, J Villar.

*Data Management Group*

DG Altman (Head), I Ahmed, S Ash, C Condon, M Mainwaring, D Muninzwa, MF da Silveira, E Staines Urias, L Walusuna, S Wiladphaingern.

*Ultrasound Group*

AT Papageorghiou (Head), L Salomon (Senior external advisor), M Buckle, N Jackson, A Mitidieri, S Munim, H Mwangudzah, R Napolitano, T Norris, J Sande, J Shah, G Zainab.

*Anthropometry Group*

L Cheikh Ismail (Head), WC Chumlea (Senior external advisor), J Kizidio, B Monyepote, F Puglia, M Salim, R Salam, VI Carrara.

*Laboratory Group*

R Craik (Head), D Alam, Y Guman, J Kilonzo, A Min, V Ngami, I Olivera, G Deutsch.

*Neonatal Group*

ZA Bhutta (Head), E Bertino, F Giuliani, R Uauy.

*Environmental Health Group*

B Eskenazi (Head), J Villar.

*Neurodevelopment Group*

A Stein (Head), M Fernandes (Coordinator), A Abubakar, J Acedo, L Aranzeta, L Cheikh Ismail, F Giuliani, D Ibanez, SH Kennedy, M Kihara, E de Leon, CR Newton, S Savini, A Soria-Frisch, J Villar, K Wulff.

Participating countries and local investigators

*Brazil*: FC Barros (Principal Investigator), M Domingues, S Fonseca, A Leston, A Mitidieri, D Mota, IK Sclowitz, MF da Silveira.

*Kenya (Kilifi)*: JA Berkley (Principal Investigator), B Kemp, H Barsosio, S Mwakio, H Mwangudzah, V Ngami, M Salim, A Seale, L Walusuna.

*Kenya (Nairobi)*: M Carvalho and W Stones (Co-Principal Investigators), D Muninzwa, J Kilonzo, J Kizidio, R Ochieng, J Sande, J Shah.

*Pakistan*: Z. Bhutta and S Munim (Co-Principal Investigators), I Ahmed, D Alam, A Raza, R Salam and G Zainab.

*South Africa*: S Norris (Principal Investigator), Y Guman, T Lephoto, S Macauley, L Malgas.

*Thailand*: F Nosten (Principal Investigator), N Jackson, R McGready, A Min, VI Cararra, S Wiladphaingern.

*UK*: SH Kennedy (Principal Investigator), S Ash, M Baricco, A Capp, L Cheikh Ismail, R Craik, S Hussein, A Laister, A Lambert, T Lewis, E Maggiora, R Napolitano, T Norris, AT Papageorghiou, B Patel, F Puglia, F Roseman, S Roseman, M Sharps, A Varalda, R Carew.
