# Supplementary material for: Machine learning for accurate estimation of fetal gestational age based on ultrasound images
Source: NPJ Digit Med. 2023 Mar 9;6:36. doi: 10.1038/s41746-023-00774-2 (PMC9998590; doi:10.1038/s41746-023-00774-2)
Supplement: Supplementary file 2 — REPORTING SUMMARY [file 41746_2023_774_MOESM2_ESM.pdf]

nature portfolio

Corresponding author(s): Prof Aris Papageorghiou

Last updated by author(s): Nov 22, 2022

Reporting Summary

Nature Portfolio wishes to improve the reproducibility of the work that we publish. This form provides structure for consistency and transparency in reporting. For further information on Nature Portfolio policies, see our Editorial Policies and the Editorial Policy Checklist.

Statistics

For all statistical analyses, confirm that the following items are present in the figure legend, table legend, main text, or Methods section.

n/a	Confirmed	
☐	☒	The exact sample size (*n*) for each experimental group/condition, given as a discrete number and unit of measurement
☐	☒	A statement on whether measurements were taken from distinct samples or whether the same sample was measured repeatedly
☐	☒	The statistical test(s) used AND whether they are one- or two-sided<br>*Only common tests should be described solely by name; describe more complex techniques in the Methods section.*
☒	☐	A description of all covariates tested
☒	☐	A description of any assumptions or corrections, such as tests of normality and adjustment for multiple comparisons
☐	☒	A full description of the statistical parameters including central tendency (e.g. means) or other basic estimates (e.g. regression coefficient) AND variation (e.g. standard deviation) or associated estimates of uncertainty (e.g. confidence intervals)
☒	☐	For null hypothesis testing, the test statistic (e.g. *F*, *t*, *r*) with confidence intervals, effect sizes, degrees of freedom and *P* value noted<br>*Give P values as exact values whenever suitable.*
☒	☐	For Bayesian analysis, information on the choice of priors and Markov chain Monte Carlo settings
☒	☐	For hierarchical and complex designs, identification of the appropriate level for tests and full reporting of outcomes
☒	☐	Estimates of effect sizes (e.g. Cohen's *d*, Pearson's *r*), indicating how they were calculated

Our web collection on statistics for biologists contains articles on many of the points above.

Software and code

Policy information about availability of computer code

Data collection	The data used is from the INTERGROWTH-21st project, and the data collection protocol is specified in the papers referenced by [25],[27] and [28]. Clinical ultrasound data were captured, as reported, by a commercially available ultrasound machine (Philips HD-9, Philips Ultrasound, Bothell, WA, USA) with curvilinear abdominal transducers (C5-2, C6-3 and V7-3). All documentation, protocols, data collection forms, and electronic transfer strategies are also freely available on the INTERGROWTH-21 st website https://www.intergrowth21.org.uk/protocol.aspx?lang=1
Data analysis	In performing our analysis, we used the following open source software:<br>Python version 3.7<br>PyTorch version 1.13.0<br>Matplotlib version 3.6.2<br>Numpy version 1.11.3<br>Seaborn version 0.12.1<br>Pandas version 1.5.1

For manuscripts utilizing custom algorithms or software that are central to the research but not yet described in published literature, software must be made available to editors and reviewers. We strongly encourage code deposition in a community repository (e.g. GitHub). See the Nature Portfolio guidelines for submitting code & software for further information.

Data

Policy information about availability of data

All manuscripts must include a data availability statement. This statement should provide the following information, where applicable:

- Accession codes, unique identifiers, or web links for publicly available datasets
- A description of any restrictions on data availability
- For clinical datasets or third party data, please ensure that the statement adheres to our policy

All documentation, protocols, data collection forms, and clinical tools are freely available on the INTERGROWTH-21st website (https://www.intergrowth21.org.uk). Ultrasound images cannot be made available due to ethical approval restrictions.

Human research participants

Policy information about studies involving human research participants and Sex and Gender in Research.

Reporting on sex and gender	Newborn sex is reported in demographics (Table 1).
Population characteristics	Population characteristics for the two datasets can be found in Table 1. This includes maternal age, height, weight and BMI. Gestational age at first ultrasound scan is also reported. Proportion of nulliparous women and rates of pre-eclampsia, preterm delivery, average birth weight of term infants and infants born at term weighing below 2500g. Newborn sex is also reported.
Recruitment	Recruitment is detailed in the Methods section for both INTERGROWTH-21st and INTERBIO-21st datasets, including inclusion and exclusion criteria.
Ethics oversight	The project was approved by the Oxfordshire Research Ethics Committee C (reference: 08/H0606/139); all the pregnant women enrolled gave informed written consent.

Note that full information on the approval of the study protocol must also be provided in the manuscript.

Field-specific reporting

Please select the one below that is the best fit for your research. If you are not sure, read the appropriate sections before making your selection.

☒ Life sciences ☐ Behavioural & social sciences ☐ Ecological, evolutionary & environmental sciences

For a reference copy of the document with all sections, see nature.com/documents/nr-reporting-summary-flat.pdf

Life sciences study design

All studies must disclose on these points even when the disclosure is negative.

Sample size	Our study retrospectively analysed ultrasound data collected as part of the INTERGROWTH-21st and INTERBIO-21st studies. All available images of the relevant planes (head circumference, abdominal circumference and femur length) were included. Not all patients had available images for all three planes and there MultiPlane includes only those that did. This is explained in the manuscript and in the footer for Table 2b (all patients in Table 2a had all three planes available).
Data exclusions	All available data was included in the analysis.
Replication	The model was developed on the INTERGROWTH-21st dataset, images were randomly assigned to training, validation or test set on a per fetus basis (randomisation using Python version 3.7). The model was then tested on an independent test set, which was unseen by the model during training. The model was then tested on data from INTERBIO-21st which acted as external validation. During the testing stages the model was blinded to the ground truth. We report results from the internal validation on INTERGROWTH-21st data as well as the results of the external validation using INTERBIO-21st.
Randomization	The INTERGROWTH-21st dataset was split by randomisation into training, validation and testting sets on a per fetus basis. No covariates were used to inform randomisation.
Blinding	The model was blinded to the ground truth (gestational age determined by the gold standard method) during the testing phase on the internal validation set from INTERGROWTH-21st and INTERBIO-21st. This is detailed in the manuscript.

Reporting for specific materials, systems and methods

We require information from authors about some types of materials, experimental systems and methods used in many studies. Here, indicate whether each material, system or method listed is relevant to your study. If you are not sure if a list item applies to your research, read the appropriate section before selecting a response.

Materials & experimental systems

n/a	Involved in the study
☒	☐ Antibodies
☒	☐ Eukaryotic cell lines
☒	☐ Palaeontology and archaeology
☒	☐ Animals and other organisms
☐	☒ Clinical data
☒	☐ Dual use research of concern

Methods

n/a	Involved in the study
☒	☐ ChIP-seq
☒	☐ Flow cytometry
☒	☐ MRI-based neuroimaging

Clinical data

Policy information about clinical studies

All manuscripts should comply with the ICMJE guidelines for publication of clinical research and a completed CONSORT checklist must be included with all submissions.

Clinical trial registration	This observational study was not registered.
Study protocol	https://www.intergrowth21.org.uk/protocol.aspx?lang=1
Data collection	The training/test set was from INTERGROWTH-21st, conducted between 2009 and 2014 in Brazil, China, India, Italy, Kenya, Oman, UK, and USA. The external validation data were from the INTERBIO-21st Fetal Study, conducted between 2012 and 2019, at six sites in Pelotas (Brazil), Nairobi (Kenya), Karachi (Pakistan), Soweto (South Africa), Mae Sot (Thailand) and Oxford (UK),
Outcomes	The outcome was the gestational age at the time of the index ultrasound scan.
